# Supplementary material for: Extraordinary siblings: Mole rats, marmosets, and Radcliffe-Brown
Source: PLoS One. 2025 Mar 28;20(3):e0319385. doi: 10.1371/journal.pone.0319385 (PMC11952269; doi:10.1371/journal.pone.0319385)
Supplement: S2 File — (DOCX) [file pone.0319385.s002.docx]

Table A1 below gives information for the *Brothers Karamazov Game, Version 3*, in which Ivan and Alyosha may have multiple opportunities to help Dmitri. The table shows payoffs in a Repeated Prisoner’s Dilemma Game involving G, a Tit For Tat conditional nepotist, and H, a “cheater.” The game is played for at least one round. On any round, the probability of playing another round is *w*, so the expected number of rounds played is 1+*w*+*w*^2^ +*w*^3^+… or 1/(1-*w*). (See the main text for more information about table organization and calculations, and for references.)

When Ivan, the more able of the two active players, is H, he offers to match Alyosha in helping Dmitri (with contributions scaled to abilities), but he always reneges on his promise, leaving Alyosha as the sole helper. When Ivan or Alyosha is G, he plays a Tit For Tat strategy, making a contribution on the first round, and then on any subsequent rounds matching what the other player did on the preceding round. When Alyosha is H he plays Tit For Tat, the same as when he is G.

TABLE A1

| Ivan  α_I_ | Alyosha  α_A_<α_I_ | Dmitri  α_D_=0 | frequency | $\Delta$G | $\Delta$H |
| --- | --- | --- | --- | --- | --- |
| G | G | G | $p^{2}+\frac{1}{4}pq$ | (α_I_+α_A_)·(b-c)  /(1-w) | 0 |
| G | G | H | $\frac{1}{4}pq$ | -(α_I_+α_A_)·c  /(1-w) | (α_I_+α_A_)·b  /(1-w) |
| G | H | G | $\frac{1}{4}pq$ | ((α_I_+α_A_)·b-α_I_c)  /(1-w) | -α_A_c  /(1-w) |
| G | H | H | $\frac{1}{4}pq$ | -α_I_c  /(1-w) | ((α_I_+α_A_)b-α_A_c) /(1-w) |
| H | G | G | $\frac{1}{4}pq$ | α_A_b -α_A_c | 0 |
| H | G | H | $\frac{1}{4}pq$ | -α_A_c | α_A_b |
| H | H | G | $\frac{1}{4}pq$ | α_A_b | -α_A_c |
| H | H | H | $q^{2}+\frac{1}{4}pq$ | 0 | α_A_b-α_A_c |

Solving for c/b< *r_G_* gives

r_G_$=\frac{\frac{1}{2}{}_{I}+\frac{1}{2}{}_{A}-\frac{1}{2}(1-w)(p-q){}_{A}}{{}_{I}+\frac{1}{2}{}_{A}-\frac{1}{2}(1-w)(p-q){}_{A}}$ Condition A1

where *q*=1-*p*. For *p*=1 this reduces to

r_G_$=\frac{\frac{1}{2}({}_{I}+w_{A})}{{}_{I}+\frac{1}{2}w_{A}}$ Condition A2

This is Condition 4 in the main text, the condition for a population of G to be resistant to invasion by a cheater, H. Note that for *w*<1 and α_A_>0, *r_G_* is a *decreasing* function of *p*. The more cheaters there are in the population (the smaller *p*-*q*), the worse cheaters do relative to conditional nepotists. For example, let α_I_ =α_A_=1.When *w*=0 and only one round is played, with *p* equal to 0, .5, or 1, *r_G_* is equal to .75, .67, or .5 respectively. And with *w*=.5 and an expected two rounds of play, with *p* again equal to 0, .5, or 1, *r_G_* is equal to .71, .67, or .60 respectively. In other words, G is more strongly favored at low than at high frequencies. A G satisfying Condition A2 can readily invade a population of cheaters, and even a very generous G – more generous than implied by Condition A2 – can increase to intermediate frequencies.

Above we consider just one variety of cheater: H cheats when, as Ivan, his ability exceeds that of the other player and he stands to bear most of the cost of repeated conditional nepotism. (In the knife-edge case of equal abilities, H as Ivan cheats with probability .5.) When H is Alyosha, and the other party stands to bear most of the cost, he plays Tit-For-Tat (including in the knife-edge case). Alternative versions of a cheater strategy, either cheat-as-Alyosha or cheat-as-Ivan-or-Alyosha, are less invasive.
