# Supplementary material for: Extraordinary siblings: Mole rats, marmosets, and Radcliffe-Brown
Source: PLoS One. 2025 Mar 28;20(3):e0319385. doi: 10.1371/journal.pone.0319385 (PMC11952269; doi:10.1371/journal.pone.0319385)
Supplement: Attachment — Submitted filename: reresubmit reviewers my response.docx [file pone.0319385.s004.docx]

Response to Reviewers.

The following is a set of responses (in italics) the points raised by the academic editor and two reviewers (plain text).

Editor’s comments (excerpt)

Both reviewers felt that the paper had value, as do I. However, both reviewers raised some concerns that need to be addressed before this manuscript could be accepted. I will not reiterate all their comments here, but I do want to highlight the comment(s) about the presentation of the mathematical models and how they should be presented in a more accessible way. This is important not just to help reviewers assess the model, but crucially to help readers who aren’t math modelers follow your logic. As Reviewer 1 notes, readers will understand your argument better if you can break down the math a bit more for them. And the better that readers can understand your argument, the more they’ll cite your arguments. As such, I request that you do more to walk readers through the model, and how the equations were derived, with or without numerical examples, to make the logic clearer.

*To help readers follow the argument, the Methods section now begins with an annotated list of subsections, laying out the different pieces of the argument to come and how they hang together, and highlighting the most important conclusions. The section concludes with a summary; I’ve added some numbers here in addition to the preceding algebra. The Results section presents a purely verbal version of the argument, first in a public goods framework (spelled out more explicitly than before), and then in multi-level selection and inclusive fitness frameworks.*

Both reviewers also noted that you can do more in the introduction to set up your main takeaway (Reviewer 1) and to distinguish this work from your previous work (Reviewer 2). PLOS ONE publishes replications and does not rely on novelty, but more readers will be excited about this paper if you can show (and show early) why this paper is novel and worth reading beyond what you’ve already published.

*These issues are covered in the responses to Reviewers 1 and 2 below.*

In addition to the reviewers’ comments, I have two additional comments. First, I notice at least one reference to Jonathan Pruitt’s work. He has multiple allegations of data fabrication against him, including multiple papers being retracted after investigation and at the request of his co-authors. He has since left academia as a result of these allegations. At this point, most or all of his papers are under a cloud of suspicion, because we don’t know which of them – if any – are based on legitimate analyses of real data. This greatly weakens any argument that relies on a citation of Pruitt. Please reconsider these citations – find a different paper to make the same point.

*I have removed the citation with Pruitt as coauthor and added a citation to a more recent review (Aviles 2021) that doesn’t cite Pruitt.*

Second, in Table 1, it’s easy for readers to misunderstand your argument about effective r’s in humans. Given that this is the introduction, readers could very easily misinterpret your argument to say that (for example) sibling r=.67 with 3 siblings under normal circumstances. Instead, these effective r’s rely on the arguments that you make later in the paper – r would not be that high without it. Is there a way you can do more to drive home that this extraordinary relatedness is not a starting point, but instead is the outcome of your model? For example, can you add a quick note to where in your paper these are derived? Example: “Humans Homo sapiens (in my model)” and “reputation + norms → socially enforced nepotism (Section X.X)”. Incidentally, this is one minor way in which you can sell the importance of this paper early, related to the reviewers' point about setting up your main takeaway earlier.

*I have changed the text as suggested, and modified the table by putting some entries in blue, as explained in the text. (I hope this is OK with copy editors.)*

Reviewer #1 (excerpts)

The various formulas and associated mathematics are particularly hard to follow. I recommend that the author help readers out by plugging some numbers into those various equations and showing how they work. The numbers could come from stories about altruism toward kin. Even if the stories are cartoonish and unrealistic, they would help the reader understand the author's point.

*See above on the list and other revisions to the Methods section.*

The section on South America is very vague and general. Can the author please provide some more details, including the names of the ethnic groups mentioned? Interested readers should not have to dig up the references the author cites in order to figure out what those groups are and how their social systems work.

*There are scores of groups with relevant social systems. I have provided details for one group, the Culina of western Amazonia, that illustrate the general claims cited.*

Early in the manuscript, explain more fully what "effective r" is and how it differs from regular r.

*I have added more in the way of explanation of effective coefficients of relatedness both in the Introduction and in the explanation of Table 1.*

The idea that altruism toward kin is a public good is crucial, but it doesn't appear until the results section. I suggest explaining it somewhere in the introduction, instead. That explanation should not assume that readers know what a public good is and what the implications are when something is a public good. Explain the characteristics of public goods (low subtractability, difficult exclusion) and explain how those characteristics can lead to a collective action dilemma, also known as a social dilemma or free-rider problem.

*The revised Introduction now includes a one paragraph introduction to social dilemmas, collective action, and the free rider problem, with citation of classic work on collective action (Olson). Another paragraph covers how these might apply to kin altruism. One more paragraph discusses the article’s contribution, and introduces the concepts of socially enforced nepotism and the effective coefficient of relatedness.*

*The beginning of the Results section has been revised with further discussion of public goods, non-excludability, and kin altruism as a public good.*

Reviewer #2: The paper presents the Brothers Karamazov Game as a means for exploring solidarity between siblings. It was difficult from this paper to determine how this advances previous papers about the Karamazov game. Also, the game nicely opens up possible strategy spaces beyond existing games. However, to be convincing, I think it would require a careful analysis of evolutionary stability with a range of possible strategies.

*I note in the Introduction that this builds on, but also adds to, previous work. In the Methods section I spell this out at several points. The important, novel conclusion that conditional nepotism can invade even at low starting frequencies occurs at the end of the Methods section.*

*The article does provide an analysis of evolutionary stability with a range of possible strategies, specifically showing (in the Competing strategies subsection) how Condition 3 beats Condition 2. Not only is socially enforced nepotism workable, but (with variation in helping ability) the frequency-independent version wins out over the frequency-dependent, hard-to-get-going version.*
